# Large paraesophageal hernia in elderly patients: Two case reports of laparoscopic posterior cruroplasty and anterior gastropexy

**DOI:** 10.1016/j.ijscr.2019.10.047

**Published:** 2019-10-28

**Authors:** Wissam G. El Hajj Moussa, Simon E. Rizk, Nidal C. Assaker, Elias S. Makhoul, Elie H. Chelala

**Affiliations:** a Department of General Surgery, Division of Laparoscopic Digestive & Bariatric Surgery, Affiliated to Faculty of Sciences and Medecine at Holy Spirit University of Kaslik, Byblos, Lebanon; b Department of Gastro-Enterology, CHU-Notre Dame des Secours, Affiliated to Faculty of Sciences and Medecine at Holy Spirit University of Kaslik, Byblos, Lebanon

**Keywords:** Case report, Paraesophageal hernia, Cruroplasty, Anterior gastropexy

## Abstract

**Background:**

Paraesophageal hernia (PEH) is a rare form of hiatal hernia, which commonly occurs in elderly people. Although asymptomatic, it can be associated with severe life-threatening complications, such as gastric volvulus. Surgical treatment is reserved for symptomatic patients. Herein, we present two cases of complicated PEH that were treated with laparoscopic posterior cruroplasty and anterior gastropexy.

**Case summary:**

An 88-year old woman presented with epigastric pain, hematemesis and food intolerance for the last two days. Physical exam revealed mild abdominal distention. Chest X-ray showed a left thoracic opacity, and barium swallow images showed a mixed type III PEH. Abdominal CT-scan images confirmed the diagnosis of incomplete gastric volvulus. The patient underwent a laparoscopic hernia reduction with sac excision, posterior cruroplasty and anterior gastropexy with continuous barbed suturing. The postoperative course was uneventful, and follow-up showed complete resolution of her symptoms.

A 91-year old patient was admitted for dyspnea and fever, with vomiting and food intolerance for the last 7 days. Physical exam revealed absent sounds on both lungs. Chest X-ray showed a large left opacity. CT-scan images revealed a giant PEH with complete gastric volvulus. The patient underwent emergency laparoscopic hernia reduction and sac excision, with re-inforced posterior cruroplasty, and anterior gastropexy with continuous barbed suturing. There were no surgical complications, but the patient died on the 4^th^ day postoperatively due to respiratory failure.

**Conclusion:**

Early laparoscopic posterior cruroplasty and anterior gastropexy is a safe and effective surgical alternative for elderly patients with comorbidities, presenting with symptomatic PEH.

## Highlights

• Large paraesophageal hernia may be associated with life-threatening complications.
• Surgical treatment is reserved for symptomatic patients.
• Sac excision and the use of mesh are still debatable options.
• Cruroplasty with anterior gastropexy may be a safe effective surgical treatment in elderly patients.

## 1 Introduction

PEH is an uncommon form of hiatal hernia. It is usually discovered in elderly people, largely due to its benign and indolent course. Although the majority of patients remains asymptomatic, PEH is associated with some severe and even life-threatening complications, such as gastric volvulus from strangulation [1]. Controversies exist regarding the optimal surgical treatment, which is reserved for symptomatic patients. Complete hernia sac excision and mesh cruroplasty are still debatable subjects due to their associated complications, especially in elderly patients with comorbidities [2]. Anterior gastropexy, although not frequently performed, can be used in these critical patients. Our aim is to emphasize on the efficacy of early laparoscopic anterior gastropexy during PEH repair, as an alternative surgical treatment for elderly patients with comorbidities. This work was written in line with the SCARE criteria [3].

## 2 Case presentation

### 2.1 Case 1

**Fig. 1:**
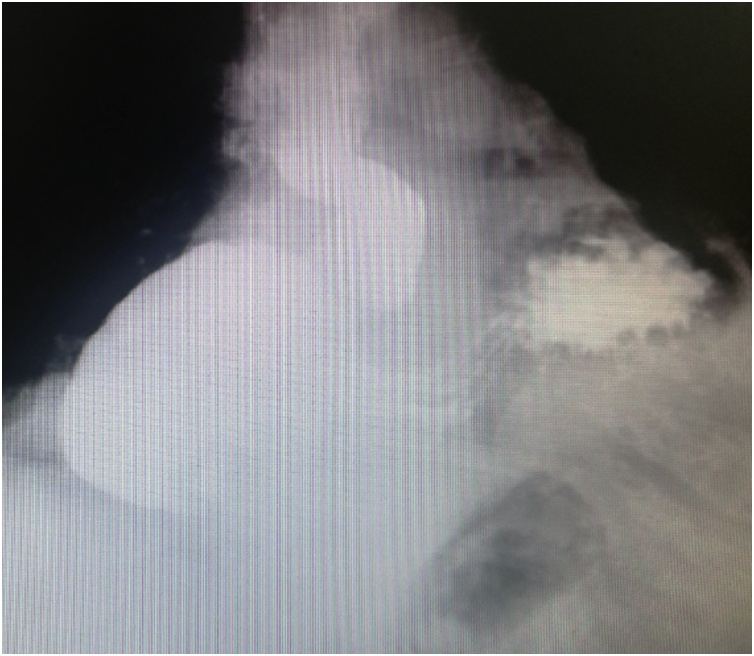
Barrium upper GI. Half the stomach body in right position.

**Fig. 2:**
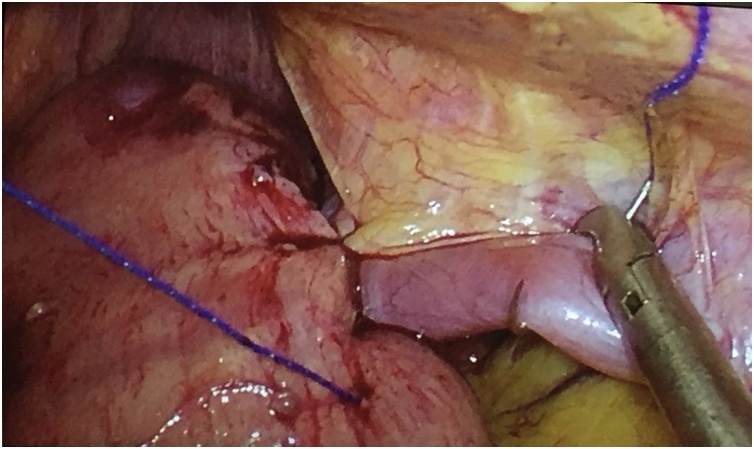
Anterior gastropexy with running unresorbable V-loc 2.0.

An 88-year old woman presented with epigastric pain, nausea and hematemesis for the last 2 days, with a recurrent history of gastro-oesophageal reflux disease (GERD), and progressive food intolerance. She had a history of well-controlled hypertension, diabetes, and chronic obstructive pulmonary disease. Physical exam upon arriving to the emergency department revealed mild abdominal distention, with non-radiating epigastric pain, without rebound tenderness or guarding, while the rest of the physical exam was normal. EKG did not reveal any abnormalities. A chest X-ray showed clear lungs with a left thoracic opacity suggestive of a large hiatal hernia. The patient was admitted and an emergency upper gastrointestinal endoscopy was performed due to persisting hematemesis, showing a mild gastritis with a bleeding mucosal lesion in the migrated stomach body that was treated with hemoclips. Barium upper gastrointestinal series showed half the gastric body above the diaphragm in the right thorax, while the Gastro-oesophageal junction was also herniating into the chest, suggesting a type III mixed PEH. The distal oesophagus had a tortuous appearance, suggestive of a probable motor dysfunction. An abdominal CT-scan with IV injection confirmed the diagnosis of incomplete gastric volvulus associated with the PEH (Fig. 1). Due to persistent epigastric pain and food intolerance, the patient underwent a laparoscopic trans-hiatal hernia and volvulus reduction, with total excision of the hernia sac. Due to the patient’s advanced age and comorbidities, we performed a posterior cruroplasty without mesh reinforcement, associated to an anterior gastropexy with continuous running suture with barbed non-resorbable 2/0, anchoring 3/4^th^ of the stomach to the anterolateral abdominal muscles (Fig. 2). The patient tolerated the procedure very well, and had an uneventful 5 days hospital stay with progressive mixed diet. She was seen at 1 and 6 months of follow-up, confirming the complete resolution of her abdominal symptoms, without GERD and a much better food tolerance.

### 2.2 Case 2

**Fig. 3:**
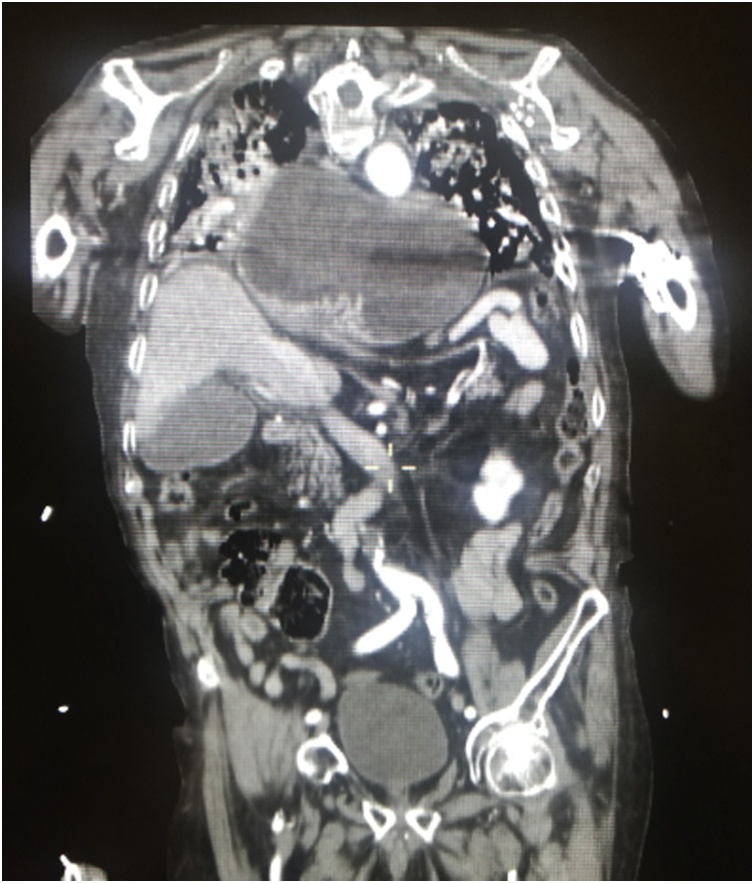
Abdominal CT. Complete intramediastinal volvulus.

**Fig. 4:**
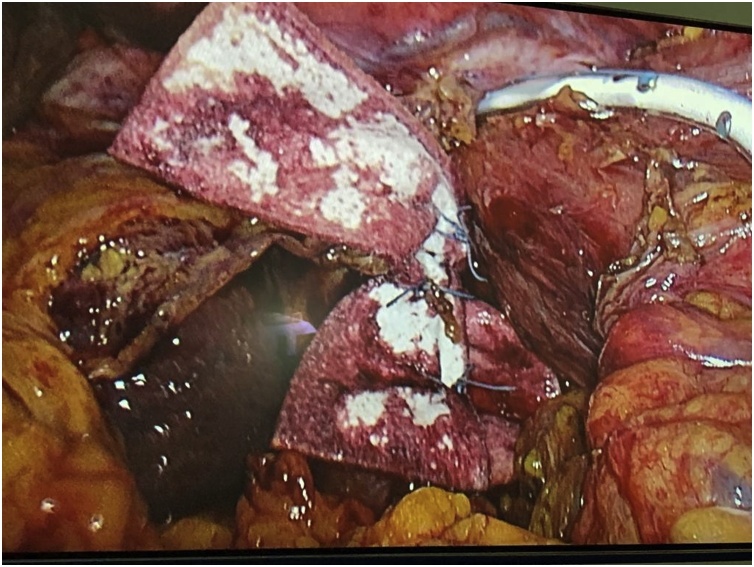
Cruroplasty reinforced with ePTFE pledgets.

A 91-year old man presented with severe dyspnea and fever for the last two days. He had a history of controlled diabetes and hypertension with heart failure. He notes persisting vomiting and food intolerance for the last 7 days. Physical examination revealed absent sounds on both lungs, with a normal abdominal exam. CXR revealed bilateral pleural effusions, left pneumonia, with a left thoracic opacity, suggestive of a large PEH. He was admitted on midcare cardiology unit for medical conservative treatment including a nasogastric decompressive tube, resuscitation and intra-venous antibiotics. A thoraco-abdominal CT-scan showed a giant paraesophageal hernia with complete intramediastinal gastric volvulus (Fig. 3). After a multidisciplinary discussion, the surgical volvulus reduction was approved and consented, as the mainstay treatment for the patient. The family members were informed and consented on the potential risks and benefits of the procedure. The patient underwent an emergency laparoscopic abdominal trans-hiatal hernia reduction, including complete sac excision, which was tightly adherent to the posterior mediastinum and needed a perioperative guided gastroscopy for the oesogastric junction, with placement of a decompressive nasogastric tube. After complete gastric reduction and sac excision we performed a posterior cruroplasty reinforced with two ePTFE large strips (Fig. 4). We also performed an anterior gastropexy with continuous barbed running suture 2/0. A mediastinal drain 16Fr. was placed. The patient was hemodynamically stable throughout the whole procedure. The patient was placed in the intensive care unit, where he was stable without any surgical complication since both drain and abdomen were clear. Unfortunately, he developed an acute respiratory failure syndrome, and died on the 4^th^ post-op. day.

## 3 Discussion

PEH comprises approximately 5 % of all hiatal hernias. The median age at presentation is 65–75 years. Most patients with PEH are asymptomatic, and many of them have non-specific complaints, such as epigastric or chest pain and vomiting, arising from obstruction. Other findings such GERD, iron-deficiency and anaemia may be also present [4]. Rarely, patients with PEH present with strangulation of the stomach, due to acute gastric volvulus, requiring emergency surgical repair, similar to our 2nd clinical case.

Prompt surgical treatment is recommended for patients with symptomatic large PEHs. However, as the majority of large PEHs occurs in the elderly population, with additional comorbidities, surgical treatment is often associated with per and postoperative risks [5]. Controversies exist in many aspects of the management of PEH, and physicians are confronted with queries regarding the optimal treatment strategies. Complete sac excision was shown to be associated with decreased recurrence rates [6]. Some authors suggest partial sac excision, especially when the sac is densely adherent to mediastinal structures, to avoid bleeding and nerve injuries [7]. Controversy exists regarding the use of mesh during PEH repair. Mesh cruroplasty has been associated with lower recurrence rates compared to suture cruroplasty alone [8,9], although some suggest comparable outcomes between the two techniques [10,11]. Nevertheless, severe complications may be associated with the use of prosthetic mesh, such as mesh infection, oesophageal erosions and stenosis [4]. Moreover, long-term follow-up of patients treated with suture cruroplasty showed similar quality of life when compared to those treated with mesh hernioraphy, even in the presence of radiological recurrence [12], the reason why we opted for a successful hernioraphy with or without large strips reinforcement in our 2 cases. As a result, the use of mesh during large PEH repair is not standardized, especially in high-risk patients, and depends on surgical findings and surgeon’s preferences. A fundoplication is usually performed during PEH repair; it has been shown that fundoplication during PEH repair was associated with less postoperative reflux and esophagitis, as well as decreasing recurrence rate of PEH, by anchoring the cardia below the diaphragm [13,14]. Nonetheless, some authors showed that fundoplication was associated with postoperative dysphagia, and thus not recommending it as a routine procedure [15], especially in elderly patients.

Laparoscopic anterior gastropexy has been described as an alternative treatment for PEH in high operative risk patients. A study evaluated 8 high-risk patients undergoing laparoscopic PEH repair with anterior gastropexy utilising interrupted transparietal suturing, and showed excellent results during a 48-month follow-up [16]. A recent study by Arevalo et al. evaluated 13 patients with a median age of 84years, with several comorbidities, treated urgently with PEH repair for obstructive symptoms. All patients underwent laparoscopic cruroplasty and anterior gastropexy, and were symptom-free during follow-up [17]. Moreover, pledgets’s use was described in a recent study by Weitzendorfer et al. for the reinforcement of hiatal sutures. They concluded that their use was safe and effective, and associated with lower recurrence rates [18]. In our experience, the anterior gastropexy was facilitated by the use of a barbed unresorbable running suture along the ¾ great curvature fixed laterally. The 2 cm large ePTFE strips reinforced the cruroplasty and helped in a better solid reapproximation of the large hiatus, thus reducing the potential risk of mesh migration. Both studies considered laparoscopic anterior gastropexy safe and effective in elderly patients with high operative risks. They concluded that it should be considered earliest as a surgical alternative for PEH in elderly patients with comorbidities, presenting with obstructive symptoms.

## 4 Conclusion

PEH in elderly comorbid patients may become life threatening, therefore the earliest diagnosis and urgent appropriate surgical repair should be proposed, in order to minimize operative time, and postoperative complications. Laparoscopic pledget’s reinforced cruroplasty with anterior gastropexy might be a safe and effective surgical alternative in these patients.

## Sources of funding

This work has no financial resources or any sponsor involvement.

## Ethical approval

This case report did not require any ethical approval and the patient and their families consented for its publication.

## Consent

The first patient consented for submitting her case for publication. Consent in the second case was obtained from the patient’s family.

## Author’s contribution

**Elie Chelala**: Operating surgeon and senior supervisor in finalizing and editing the article.

**Elias Makhoul**: Gastroenterologist who performed the per-opertaive endoscopy and editing coordinator.

**Wissam El Hajj Moussa**: PGY-4 visceral and digestive surgery at Notre Dame de Secours-Jbeil and Holy-Spirit university of Kaslik. 1^st^ author writing the manuscript.

**Simon Rizk**: PGY-3 visceral and digestive surgery at Notre Dame de Secours-Jbeil and Holy-Spirit university of Kaslik. Cooperating for the writing of the manuscript.

**Nidal Assaker**: PGY-3 visceral and digestive surgery at Notre Dame de Secours-Jbeil and Holy-Spirit university of Kaslik. Cooperating for the writing of the manuscript.

## Registration of research studies

Not applicable in this case.

## Guarantor

Prof. Elie Chelala.

## Provenance and peer review

Commissioned, externally peer-reviewed.

## Declaration of Competing Interest

Pr. Elie Chelala declare being a consultant surgeon for Medtronic USA but has no conflict of interest in this article, as well as all the authors mentioned.
